# Factors associated with caregiver burden in a child and adolescent psychiatric facility in Lagos, Nigeria: a descriptive cross sectional study

**DOI:** 10.1186/1471-2431-11-110

**Published:** 2011-12-12

**Authors:** Mobolaji U Dada, Niran O Okewole, Oluyemi C Ogun, Mashudat A Bello-Mojeed

**Affiliations:** 1 Ekiti State University Teaching Hospital, Ekiti, Nigeria; 2 Neuropsychiatric Hospital, Aro Abeokuta, Nigeria; 3 Federal Neuropsychiatric Hospital, Yaba, Lagos, Nigeria; 4 Federal Neuropsychiatric Hospital, Yaba, Lagos, Nigeria

## Abstract

**Background:**

Definitions of burden of care stress the effect of the patient's mental illness on the family. There are generally very few studies in this environment on caregiver burden in child/adolescent mental ill-health. This study aimed to identify patient and caregiver characteristics that are associated with caregiver burden.

**Method:**

Caregivers of patients attending the Child and Adolescent Clinic of the Neuropsychiatric Hospital, Yaba, Lagos [n = 155] were consecutively recruited over a one-month period. The caregivers were administered a sociodemographic questionnaire, the General Health Questionnaire, Zarit Burden Interview, and the Columbia Impairment Scale. Scoring on the Children's Global Assessment Scale was done by clinicians.

**Results:**

Most caregivers observed in this study were females (80.5%), with mothers of the patients accounting for 78% of all the caregivers. A higher percentage of the patients were males (52.8%). Moderate to severe/severe burden was recorded among 25.2% of caregivers. Factors associated with caregiver burden were patient's level of functioning [r = 0.489, p < 0.001], psychiatric morbidity in the caregiver [r = 0.709, p < 0.001], level of impairment as assessed by the caregiver [r = 0.545, p < 0.001], and child's level of education [t = 3.274, p = 0.001]. Each one independently predicted caregiver burden.

**Conclusion:**

The study reveals a high level of burden among the caregivers of children and adolescents with mental health problems.

## Background

Burden of care has been defined as "the presence of problems, difficulties or adverse events which affect the life (lives) of the psychiatric patient's significant other(s)"[1]. These occur as a result of the challenges involved in caring for the mentally ill patient. Various definitions have been given, all stressing the effect of the patient's illness on the family, or the impact that the patient's illness has on the family's daily routine, or health [2]. In addition to the emotional, psychological, physical and economic impact, the concept of 'burden of care' involves subtle but distressing notions such as shame, embarrassment, feelings of guilt and self-blame [3].

Previous studies have provided consistent evidence that the care-givers of persons with chronic mental illness suffer from a number of significant stresses and moderately high levels of burden [4]. The care-giver is usually a relative of the ill person and the care given is invariably continuous. He or she often has additional responsibilities in the family and many of the care recipients do not acknowledge or even recognise the assistance and help they are receiving. The care is given because of emotional bonding, duty, guilt and/or the lack of other available services in the community [4].

Dimensions of the burden of caregiving include the symptom-specific burden impact of the disability associated with the illness itself, both in terms of demands for assistance and supervision, and regarding the potential stigma associated with the illness; the social burden impact on family and other social relationships; the emotional burden impact on mental and emotional well-being; and the financial burden impact on work and the general financial costs of care-giving [4]. Parents of children with mental health disorders are more likely than other parents to cut work hours, to quit work, and to spend more time arranging their child's care [5], the time involved in caring for chronically ill children leading to lost opportunities [6]

Parental physical health and the child's educational level have been reported as strong predictors of caregiver burden [7]. Other predictors include vocational/occupational status of the mothers, child's symptomatology and impairment, parental mental health problems, male patients with schizophrenia, intense psychotic symptoms, cultural and ethnic factors [3,8]. Studies have shown that older parents were disturbed by the cognitive dimensions of burden, while younger parents were distressed by their offspring's behaviour [9].

A decade ago, Angold et al [2] reported that little attention had been paid to the parental burden resulting from caring for children and adolescents with psychiatric disorders, even in developed countries. In the ensuing years this situation changed somewhat but in developing countries there still remains a serious dearth of information regarding caregiver burden, existing studies focusing on caregiving in the adult psychiatric population [10]. The implication of this is that there is a hiatus in available evidence on which policy and health care planning can be based. This diminishes the quality of service available to the caregiver and by extension the patient.

This study, therefore, aimed to determine the demographic characteristics of patients and their caregivers which are associated with burden, as well as the impact of patient diagnosis, level of functioning/impairment, and presence of psychopathology in the caregiver.

## Method

### Location of the Study

The study was carried out at the Harvey Child and Adolescent Clinic of the Federal Neuropsychiatric Hospital, Yaba, Lagos. The Centre was established in May 1999 to cater for children and adolescents with mental health problems. Patients are seen from within and outsde Lagos State.

### Sampling

Over a one month period [July 2008], caregivers of patients attending the outpatient clinic were approached to be recruited into the study. For inclusion, a subject had to be a primary caregiver, that is, living with the patient, responsible for monitoring and funding the patient's treatment and general welfare, and the one usually called upon in emergencies. Also, the patient must have had the illness for a duration not less than 6 months. Those who did not meet these criteria were excluded. Diagnosis was made either by a consultant or senior registrar and was based on DSM-IV criteria. A total of 155 caregivers, one for each patient, consented to take part in the study.

### Instruments

The caregivers were administered a sociodemographic questionnaire, the 12-item version of the General Health Questionnaire (GHQ-12), the Columbia Impairment Scale (CIS) and the Zarit Burden Interview (ZBI). Scoring on the Children's Global Assessment Scale (CGAS) was done by clinicians. GHQ-12 and the ZBI were available in Yoruba versions. The CGAS, being rated by the clinician, did not require translation. Caregivers were assisted in filling the CIS.

The Zarit Burden Interview [11] is a self administered 22-item questionnaire with a five-item response set ranging from ''never'' to ''nearly always''. 0-20 points mean little or no burden, 21-40 points mean mild to moderate burden, 41-60 points mean moderate to severe burden, 61-88 points mean severe burden. The ZBI includes factors most frequently described by caregivers as problematic, such as their physical and psychological health, finances, social life, and the relationship with the patient.

The 12-item version of the General Health Questionnaire [12], was used to screen for psychiatric morbidity in the caregivers. Each item is accompanied by four responses, typically 'not at all', 'no more than usual', 'rather more than usual' and 'much more than usual'. Scores are assigned using a binary method (0-0-1-1). The caseness threshold for GHQ-12 is a score of 3 or more. GHQ-12 is of proven validity internationally [13].

The Columbia Impairment Scale [14] was used to obtain the caregivers' assessment of impairment in the patient. The CIS is a 13 item questionnaire which can be administered or used as self-report. It is scored using a 5-point likert where 0 = 'no problem' and 4 = very big problem'. It assesses four key areas of functioning: interpersonal relations, broad psychopathology domains (e.g. anxiety, depression, behavioral problems), functioning in job or school and use of leisure time.

The Children's Global Assessment Scale, CGAS [15] is one of the most widely used measures of overall illness severity in children. It is a unidimensional (global) measure of social and psychiatric functioning. It has a single rating scale with scores ranging from 1 to 100, anchored at 10 point intervals with descriptors for each interval. It is of proven reliability and validity [16,17]. Illiterate respondents were assissted by the authors who read out the questionnaire to them.

Other variables such as clinical diagnoses were obtained from the patients' case files while socio-demographic information on patients was obtained either from the hospital records or from the caregiver.

### Ethical Consideration

The Ethics and Research Committee of Federal Neuropsychiatric Hospital, Yaba, Lagos approved the study protocol while informed consent was obtained from the caregivers after the aims and objectives of the study had been explained to them. Verbal assent was also obtained from the patients themselves. Those caregivers who showed high psychiatric morbidity in the study were offered specialist intervention.

### Data analytic procedures

Results were calculated as frequencies for categorical variables and means for continuous variables. Comparison of variables with Zarit scores was done using chi squares, analysis of variance and correlation. Significant variables were thereafter entered into stepwise multiple regression, with adjustments for patient and caregiver variables. Tests were two-tailed and level of significance was set at 95%. Statistical analysis was done using Statistical Package for Social Sciences (SPSS), version 14.

## Results

### Sociodemographic profile

A total of 159 caregivers, one for each child, were approached for the study. Of these, 155 caregivers agreed to take part in the study, giving a response rate of 97.5%. The mean age of caregivers was 41.5 years [sd 8.9]. Majority (78%) were mothers while others were either fathers, siblings, aunts, uncles or grandparents.

The mean age of patients was 12.3 years [sd 4.98], with ages ranging from 2 to 18 years. Of the patients, 52.8% were male while the rest were female. 30.9% were in Nursery/primary school, 21.5% were in secondary school while the rest were either in a vocational/special school, out of school, or had had no formal education. 12.1% had been ill for less than a year, 39.6% for 1 - 5 years while the rest had been ill for over 5 years.

### Patient Diagnoses

More than half of the patients [56.4% ] had a main diagnosis of seizure disorder. Other main diagnoses were attention deficit/hyperactivity disorder [16.2%], autism [6.8%], mood disorder [7.7%], and organic psychosis [1.7%], schizophrenia [1.7%], unspecified psychosis [6.8%] and mental retardation [2.6%]. 37.4% had comorbid conditions, mainly intellectual disability and seizure disorder while in two cases there was comorbid sickle cell disease.

### Zarit Scores

Median Zarit score was 25, with 48.4% above the median. 41.3% of caregivers had Zarit scores of 0 - 20 (little or no burden), 33.5% had scores of 21 - 40 (mild to moderate burden), while 22% had scores of 41 - 60% (moderate to severe burden). 3.2% had scores over 60 (severe burden). This last group was merged with those with 'moderate to severe burden' for analysis.

### CGAS, GHQ-12 and CIS Scores

53.8% of those assessed had CGAS scores less than or equal to 50. Mean CGAS score was 55 [sd 22.8]. Mean GHQ score was 2.5[sd 2.6], while 39.4% had GHQ Scores of 3 and above. Mean score on the Columbia impairment Scale was 16.38 [sd 14.296].

### Comparison of Variables with Zarit Score

Sociodemographic variables of the children and caregivers were compared with total Zarit scores. No significant association was found except for level of education of the child (t = 3.274, p = 0.001). Thereafter comparisons were done with burden categories.

Table 1 shows the comparison of caregiver variables with caregiver burden. None of the caregiver demographic variables showed a significant association with burden categories.

**Table 1:** Comparison of caregiver burden with caregiver variables.

Variable	Little/no	Mild/moderate	Moderate/severe	Difference
Mean age (sd)	41.6(9.2)	39.9(8.4)	43.3(8.9)	F = 0.246
				df = 2,151
				p = 0.783

Gender				χ^2 ^= 0.383
Male	14(21.9%)	9(17.3%)	8(20.5%)	df = 2
Female	50(78.1%)	43(82.7%)	31(79.5%)	p = 0.826

Relationship				χ^2 ^= 0.244
Mothers	50(78.1%)	39(75%)	29(74.4%)	df = 2
Others	9(14.3%)	13(25%)	10(25.6%)	p = 0.885

Employment status				χ^2 ^= 0.147
Employed	54(85.7%)	42(84%)	33(86.8%)	df = 2
Unemployed	9(14.3%)	8(16%)	5(13.2%)	p = 0.929

Marital status				χ^2 ^= 0.539
Married	53(82.8%)	42(80.8%)	30(76.9%)	df = 2
Not married	11(17.2%)	10(19.2%)	9(23.1%)	p = 0.764

A comparison of patient variables with categorized caregiver burden is shown in Table 2. These also showed no significant association. Patient diagnoses were classified into those with/without seizure disorder. No significant difference was observed with respect to caregiver burden. The diagnoses were further classified as those with/without psychosis. This also showed no significant association.

**Table 2:** Comparison of caregiver burden with patient variables.

Variable	Little/no	Mild/moderate	Moderate/severe	Difference
Mean age (sd)	12.2(4.7)	12.1(5.1)	12.8(5.3)	F = 1.55
				df = 2,148
				p = 0.216

Gender				χ^2 ^= 1.37
Male	29(50.9%)	24(49%)	22(61.1%)	df = 2
Female	28(49.1%)	25(51%)	14(38.9%)	p = 0.504

Level of education				χ^2 ^= 1.58
≤ Primary	45(70.3%)	41(78.8%)	31(79.5%)	df = 2
≥ Secondary	19(29.7%)	11(21.2%)	8(20.5%)	p = 0.454

Duration of illness				χ^2 ^= 1.252
< 1 year	8(12.9%)	6(11.7%)	4(11.1%)	df = 4
1 - 5 years	23(37.1%)	19(37.3%)	17(47.2%)	p = 0.881
> 5 years	31(50.0%)	26(51.0%)	15(41.7%)	

Diagnosis				χ^2 ^= 0.148
Seizure disorder	28(58.3%)	24(55.8%)	14(53.8%)	df = 2
Others	20(41.7%)	19(44.2%)	12(46.2%)	p = 0.929

Presence of psychosis				χ^2 ^= 0.148
Yes	7(14.9%)	6(14%)	3(12%)	df = 2
No	40(85.1%)	37(86%)	22(88%)	p = 0.862

Scores on the CGAS, CGAS and CIS were also compared with raw Zarit scores. All three were found to be significantly associated with burden of care: patient's level of functioning [r = 0.489, p < 0.001], psychiatric morbidity in the caregiver [r = 0.709, p < 0.001] and level of impairment as assessed by the caregiver [r = 0.545, p < 0.001].

GHQ scores, CGAS scores, CIS scores and level of education of the child were entered into stepwise multiple regression, with adjustments for age and sex of patients and caregivers, caregiver marital status, duration of child's illness, diagnosis, and presence of psychosis. The model summary (table 3) shows that all four variables predicted caregiver burden with a total explained variance of nearly 55%.

**Table 3:** Stepwise multiple regression

Model	**R**^**2**^	R^2 ^change	Standardised Coefficient, β	p value
1 GHQ	0.395	0.395	0.629	< 0.001
				
2 GHQ	0.502	0.107	0.566	< 0.001
CGAS			- 0.332	< 0.001
				
3 GHQ	0.546	0.044	0.466	< 0.001
CGAS			- 0.304	< 0.001
CIS			0.236	0.004
				
4 GHQ	0.546	0.020	0472	< 0.001
CGAS			- 0.251	0.001
CIS			0.222	0.006
Education			- 0.152	0.045

Dependent variable: Zarit scores

All models adjusted for age and gender of patients and caregivers, caregiver marital status, duration of child's illness, diagnosis, and presence of psychosis.

## Discussion

The study revealed a considerable burden experienced by the caregivers of children and adolescents attending the clinic. This burden is predicted by the presence of psychiatric morbidity in the caregiver, the level of functioning as assessed by the clinician, the degree of impairment as assessed by the caregiver, and the level of education of the child.

As in previous studies is this environment [18], a higher percentage of the patients in this study were males. The gender and age of the patient were however not significantly associated with burden of care. Neither was duration of illness.

The level of education of the patient showed a significant association, and as in a previous study [7] predicted burden of care, albeit accounting for only 2% of the variance. A lower level of patient's education predicted a higher burden of care. It is possible that children with higher educational attainment may be more mature, suffer less intellectual deficits, and therefore are capable of taking better care of themselves, thereby imposing less burden on the caregivers.

Most caregivers observed in this study were females, with mothers of the patients accounting for 78% of all the caregivers. This highlights the challenges faced by mothers. Gender and relationship with the patient, although predictors of burden in the adult population [8], were not significantly associated with burden in this study. Although female caregivers especially mothers were predominant and thus merit special attention, the study suggests that caring for a mentally ill child is burdensome on the caregiver, regardless of gender or relationship. Age, marital and employment status were also not associated with burden of care. The duration of illness and the diagnosis of the patient, including the presence or otherwise of psychosis, was not significantly associated with burden of care.

Presence of psychiatric morbidity in the caregiver was however found to predict caregiver burden, accounting for nearly 40% of the variance. This might be a bidirectional relationship in that caregivers with pre-existing psychiatric conditions might find caring for a sick child burdensome, while the burden of caring for a sick child could also precipitate psychological distress.

Available evidence suggests that children with mental health concerns are more likely to have mothers who screen positive for a mental illness [19]. This also might be reciprocal in that there might be a hereditary component to the child or adolescent's disorder, while the burden of caregiving could be a factor in the onset of psychological symptoms in the parent.

Other factors found to predict caregiver burden are the level of impairment as assessed by the clinician and the level of functioning as assessed by the caregiver. The two are related and imply that a child with more impairment or a lower level of functioning is likely to require more assistance from the caregiver in terms of activities of daily living and as such may impose a greater burden on the caregiver.

The implications of these findings are that caregivers whose children have more impairment or functional difficulties will require more support services in order to lessen the burden of caregiving. This in turn could help to prevent psychiatric morbidity in the caregiver. Child health services also need to make arrangements for attending to caregivers with preexisting mental illness as well as those who develop mental health problems in the process of caregiving. In a resource poor setting, this challenge can be enormous.

This study gives much needed information on the burden of caring for children and adolescents in a resource-constrained developing country like Nigeria. Hitherto there has been a paucity of information in this regard. It is however limited in that it involved only one centre within the country. This fact restricts the extent to which the findings are generalisable. The modest sample size is also a limitation which further studies can improve upon.

## Conclusion

Child mental health services cannot be considered adequate unless they factor in the needs of the caregivers. These include access to information, social resources, and better access to treatment for physical and psychological problems. More studies need to be conducted on various aspects of caregiver status such as strengths and needs, quality of life, treatment satisfaction, psychiatric morbidity, and coping with stigma.

## Competing interests

The authors declare that they have no competing interests.

## Authors' contributions

MUD and NOO participated in the design of the study, statistical analysis and manuscript preparation. OCO participated in study design and coordination. MAB contributed to data collection and statistical analysis. All authors read and approved the final manuscript.

## Pre-publication history

The pre-publication history for this paper can be accessed here:

http://www.biomedcentral.com/1471-2431/11/110/prepub
